# Creating Completely Both Male and Female Sterile Plants by Specifically Ablating Microspore and Megaspore Mother Cells

**DOI:** 10.3389/fpls.2016.00030

**Published:** 2016-02-01

**Authors:** Jian Huang, Ashley R. Smith, Tianyu Zhang, Dazhong Zhao

**Affiliations:** Department of Biological Sciences, University of Wisconsin–MilwaukeeMilwaukee, WI, USA

**Keywords:** completely both male and female sterile plants, flower structure, genetic ablation, gene flow, microspore and megaspore mother cells, *SOLO DANCERS*

## Abstract

Although genetically modified (GM) plants have improved commercially important traits, such as biomass and biofuel production, digestibility, bioremediation, ornamental value, and tolerance to biotic and abiotic stresses, there remain economic, political, or social concerns over potential ecological effects of transgene flow from GM plants. The current solution for preventing transgene flow from GM plants is genetically engineering sterility; however, approaches to generating both male and female sterility are limited. In addition, existing strategies for creating sterility lead to loss or modifications of entire flowers or floral organs. Here, we demonstrate that instead of the 1.5-kb promoter, the entire *SOLO DANCERS* (*SDS*) gene is required for its meiocyte-specific expression. We then developed an efficient method to specifically ablate microspore and megaspore mother cells using the *SDS* and *BARNASE* fusion gene, which resulted in complete sterility in both male and female reproductive organs in *Arabidopsis* (*Arabidopsis thaliana*) and tobacco (*Nicotiana tabacum*), but did not affect plant growth or development, including the formation of all flower organs. Therefore, our research provides a general and effective tool to prevent transgene flow in GM plants.

## Introduction

Since genetically modified (GM) plants were produced in 1983 (Caplan et al., 1983; Murai et al., 1983), the number of GM plants has been rapidly increasing yearly (Husken et al., 2010). GM trees, turf grasses, biofuel and forage crops, and ornamentals have improved commercially important traits, including biomass and biofuel production, digestibility, bioremediation, ornamental value, and tolerance to biotic and abiotic stresses (Wang and Ge, 2006; Groover, 2007; Harfouche et al., 2011; Xu et al., 2011; Wang and Brummer, 2012; Wilkerson et al., 2014); however, the approval for commercialization of GM plants is subject to complicated and stringent government regulations due to economic, political, or social concerns over potential ecological effects of transgene flow and floral-modified plantations (Goeschl and Swanson, 2003; Hills et al., 2007; Van Acker et al., 2007; Strauss et al., 2009; Lombardo, 2014).

Transgene flow from GM plants to non-GM plants and wild populations is mainly mediated by dispersal of pollen and seeds. Early studies found that the pollen-mediated gene flew from GM Roundup Ready creeping bentgrass (*Agrostis stolonifera*) occurred within 2 to 21 km (Watrud et al., 2004). The non-GM rabbitfoot grass (*Polypogon monspeliensis*) could pollinate the GM creeping bentgrass to produce transgenic intergeneric hybrid offspring, suggesting that the transgene escape is also mediated by the female part of GM plants (Zapiola and Mallory-Smith, 2012). Long distance pollen-mediated gene flow occurred between weed beets (*Beta vulgaris*) as far as 9.6 km and the resulting interfield gene flow was unavoidable (Fenart et al., 2007). Pollen migration from poplars (*Populus trichocarpa*) often went beyond 10 km (Slavov et al., 2009; DiFazio et al., 2012), indicating that similar issues happened in GM trees. Moreover, gene flow from GM crops to native populations was detected in maize (*Zea mays*), soybean (*Glycine max*), wheat (*Triticum aestivum*), and canola (*Brassica napus*; Pineyro-Nelson et al., 2009; Liu et al., 2010b; Rieben et al., 2011; Wang and Li, 2012). To overcome regulatory hurdles to field research and, ultimately, commercial uses of GM plants, a practical solution is to create sterile plants by ablating floral organs/tissues using toxic genes under control of specific promoters or by altering flowering time and floral organs via manipulating genes critical for flower development.

Strategies on making male sterility have been extensively and successfully employed to prevent the pollen-mediated transgene flow. In the male reproduction organ anther, tapetum is a layer of nutritive cells, which is required for pollen development. Therefore, genetic ablation of tapetal cells by tapetum-specific promoters driven toxic genes, such as ribonuclease *BARNASE* and diphtheria toxin fragment A (*DTA*) genes, is commonly used to create male sterility in various plants. The widely used tobacco tapetum promoter *TA29* was first employed to drive *BARNASE* to create male sterile tobacco and oilseed rape (*Brassica napus*) plants (Mariani et al., 1990). *TA29::DTA* tobacco transgenic plants are also male sterile (Koltunow et al., 1990). Since then, various male sterile plants were achieved using other tapetum or anther-specific promoters, including *A9*, *A6*, *E1*, *T72*, *PS1*, and *PsEND1* in *Arabidopsis*, rapeseed (*Brassica napus*), rice (*Oryza sativa*), and pea (*Pisum sativum*) plants (Paul et al., 1992; Hird et al., 1993; Zhan et al., 1996; DeBlock et al., 1997; Roque et al., 2007). This strategy was also applied to perennial grasses and trees. The *TAP::BARNASE* creeping bentgrass is completely pollen sterile (Luo et al., 2005). *PrMC2*, a pine male cone-specific gene, was successfully used to generate male sterile pine (*Pinus radiata*) and *Eucalyptus* (sp.) plants by driving a modified *BARNASE* gene (Zhang et al., 2012). It was recently reported that the *TA29::BARNASE* transgenic poplar constantly showed robust male sterility during a 4-year field trial (Elorriaga et al., 2014). Attempts were also made to abolish male and female fertility together. In *Arabidopsis*, *BARNASE* driven by the second intron of *AGAMOUS* resulted in ablation of stamens and carpels (Liu and Liu, 2008). Male and female sterile tobacco plants were generated by expressing *BARNASE* under control of both the tapetum promoter *p108* and the transmitting tract promoter *sp41* (Gardner et al., 2009).

In addition, manipulating genes regulating flowering time, floral meristem identify, floral organ identity, and floral organ establishment is used to abolish plant fertility. Silencing the tobacco *LEAFY* genes *NFL1* and *NFL2* resulted in plants without flowers (An et al., 2011). Tomato (*Lycopersicon lycopersicum*) *AGAMOUS* RNAi lines showed “fruit-in-fruit” phenotype, but did not produce seeds (Pan et al., 2010). Down-regulation of *APETALA3* genes *OsMADS16* and *MtNMH7* caused stamen to carpel transformation and male sterility in rice and *Medicago truncatula*, respectively (Xiao et al., 2003; Roque et al., 2013). Expression of *TFL1*, a strong floral repressing gene, led to the non-flowering phenotype in red fescue (*Festuca rubra;* Jensen et al., 2004). Moreover, overexpression of miR156 inhibited flowering in switchgrass (*Panicum virgatum*; Fu et al., 2012). Besides generating sterile plants, plastid transformation is also an excellent approach to prevent pollen-mediated transgene flow, since plastids, including chloroplasts, are maternally inherited in most plants (Ruf et al., 2007; Wani et al., 2010).

Although male sterility has been successfully achieved via different approaches in various plant species, it cannot completely prevent transgene flow. Seed development in male sterile GM plants can be rescued by the long-distance transfer of pollen from non-GM plants. The same is also true for female sterile GM plants which disperse pollen to non-GM or male sterile GM plants. Thus, completely abolishing both male and female fertility is the only fail-safe way to prevent transgene flow (Stewart, 2007). So far, approaches to generating complete both male and female sterility are limited. Moreover, existing strategies for creating male and/or female sterility lead to loss or modifications of entire flowers or floral organs (Xiao et al., 2003; Roque et al., 2007; Pan et al., 2010; An et al., 2011), which may cause potential ecological effects on biodiversity of species associated with flowers, such as insects. In economically interesting species, for example ornamentals, altered flowers may also be undesirable. Furthermore, since the remaining toxicity of BARNASE or DTA in non-target organs due to the non-specific basal activities of employed promoters often inhibits plant survival and growth (Lannanpaa et al., 2005; Wei et al., 2007), it is difficult to obtain usable sterile plants that have normal biomass and yield. Therefore, it is imperative to generate sterility in both male and female reproductive organs without affecting plant growth or modifying flower structure.

In *Arabidopsis*, the *SOLO DANCERS* (*SDS*) gene, which encodes a meiosis-specific cyclin, is required for homolog interaction during meiotic prophase I in *Arabidopsis* (Azumi et al., 2002). With normal growth and development, the *sds* mutant is both male and female sterile. RNA *in situ* hybridization analysis showed that *SDS* transcripts were specifically present in microspore mother cells (male meiocytes) in anthers and megaspore mother cells (female meiocytes) in ovules (Azumi et al., 2002). Here, we report our new approach to create complete both male and female sterility in *Arabidopsis* and tobacco by specifically ablating microspore and megaspore mother cells using the *SDS* and *BARNASE* fusion gene. Our research provides a general and effective tool to prevent transgene flow in GM plants.

## Materials and Methods

### Plant Materials and Growth Condition

*Arabidopsis thaliana* Landsberg *erecta* (L*er*) and tobacco (*Nicotiana tabacum* Petit Havana *SR1*) were used in this study. Plants were grown in Metro-Mix 360 soil (Sun-Gro Horticulture, Agawam, MA, USA) in a growth chamber under a 16-h light/8-h dark photoperiod regime at 22°C and 50% of humidity.

### Generation of Constructs and Transgenic Plants

PCR reactions (see all primers in Supplementary Table S1) were performed using Phusion High-Fidelity DNA Polymerase (New England Biolabs, Ipswich, MA, USA). The 1.5-kb promoter of the *SDS* gene (upstream of the *SDS* coding region and the 3′ non-coding region of the *SDS* adjacent gene) was amplified and cloned into the pENTR/D-TOPO vector (Invitrogen, Grand Island, NY, USA) to generate pENTR-*SDS.* The *SDS* genomic fragment from the beginning of the 1.5-kb promoter region to the last exon was introduced into the pENTR/D-TOPO vector to generate pENTR-*SDS::SDS.* The *BARSTAR* gene amplified from the pABGCZ vector (Zhang et al., 2012) was introduced to the pEarleyGate303 vector at the Nsi site to generate pEarleyGate303-*BARSTAR.* An XhoI site was introduced between BglII and XbaI sites right after attR2 to generate pEarleyGate303-*BARSTAR(XhoI).* The *BARNASE* fragment amplified from pABGCZ was cloned into pEarleyGate303-*BARSTAR(XhoI)* using the XhoI and XbaI sites to generate pEarleyGate303-*BARSTAR-BARNASE.* Using the Gateway LR recombinase II enzyme mix (Invitrogen, Grand Island, NY, USA), *SDS::GUS*, *SDS::BARNASE*, *SDS::SDS-GFP*, and *SDS::SDS-BARNASE* binary vectors were generated between pENTR-*SDS* and pGBW3-GUS, pENTR-*SDS* and pEarleyGate303-BARSTAR-BARNASE, pENTR-*SDS::SDS* and pGBW4-GFP, as well as pENTR-*SDS::SDS* and pEarleyGate303-BARSTAR-BARNASE, respectively.

The floral dip method was used to generate *Arabidopsis* transgenic plants (Clough and Bent, 1998). Transformants of *SDS::GUS* and *SDS::SDS-GFP* were screened on 50 μg/mL of kanamycin and 25 μg/mL of hygromycin. Transformants of *SDS::BARNASE* and *SDS::SDS-BARNASE* were screened on 1% of Basta (PlantMedia, Lubbock, TX, USA).

Tobacco transformation was performed as described previously (Curtis et al., 1995). Briefly, leaf disks were inoculated with the *Agrobacterium* strain GV3101 containing the *SDS::SDS-BARNASE* binary vector and cultured for 1 day in the dark, followed by 2 days under light. Then, leaf disks were screened on shoot and root selection medium containing 4% of Basta. The regenerated seedlings were transferred into soil and sprayed with 4% of Basta solution one week later. The surviving plants were used for further analyses.

### Pollen Staining and Anther Semi-Thin Sections

To examine pollen viability in *Arabidopsis* plants, Alexander pollen staining was carried out as described previously (Zhao et al., 2002). Briefly, main inflorescences were collected when 1–4 flower(s) were opened. Inflorescences were fixed for 24 h in the fixative containing methanol, 60 mL; chloroform, 30 mL; distilled water, 20 mL; picric acid, 1 g; and HgCl_2_, 1 g. After transferring through 70, 50, and 30% ethanol series (30 min in each change), inflorescences were finally incubated with water. Inflorescences were them transferred into the staining solution (ethanol 95%, 10 ml; malachite green, 10 mg; acid fuchsin, 50 mg; orange G, 5 mg; phenol, 5 g; glacial acetic acid, 2 ml; glycerol, 25 ml; and distilled water 50 ml) and kept at 50°C for 48 h. Individual anthers were dissected out from flowers and then mounted on the glass slides together with the staining solution for observation. Mature anthers from tobacco plants were collected and analyzed using the same method. Pollen grains were released from anthers before imaging.

Semi-thin sectioning was performed as described in our previous studies (Zhao et al., 2002; Jia et al., 2008). Briefly, dissected floral buds were fixed in 2.5% (vol/vol) glutaraldehyde in 0.1 M HEPES (*N*-2-Hydroxyethyl piperazine-*N*_-2-ethanesulfonic acid) buffer (pH 7.2) and 0.02% TritonX-100 overnight at room temperature. Samples were washed three times for 15 min each in 0.1 M HEPES buffer with 0.02% Triton X-100 and then fixed in 1% OsO4 overnight at room temperature. Samples were then dehydrated in a graded acetone series (10% increments) for 60 min each. Infiltration started with 20% Spurr’s resin and then 40, 60, and 80% Spurr’s resin every 3 h. Samples were transferred to 100% Spurr’s resin three times for 24 hours each. Samples were finally embedded in 100% Spurr’s resin and polymerized at 60°C overnight. Semi-thin (0.5 μm) sections were made using an Ultracut E ultramicrotome (Reichert–Jung) and were stained with 0.25% Toluidine Blue O.

### GUS Staining Assay

Histochemical GUS staining assay was performed as previously described (Liu et al., 2010a).

Briefly, tissues were collected and fixed for 1 h in 90% acetone at –20°C. After washing tissues in washing buffer [0.1 M phosphate (pH 7.0), 10 mM EDTA, and 2 mM K_3_Fe(CN)_6_] twice for 5 min under the vacuum, the drained tissues were transferred into the GUS staining buffer [0.1 M phosphate (pH 7.0), 10 mM EDTA, 1 mM K_3_Fe(CN)_6_, 1 mM K_4_Fe(CN)_6_⋅3H_2_O, and 1 mg/ml X-GLUC)] and incubated overnight at 37°C. GUS-stained tissues were then fixed in a 3:1 mixture of ethanol and acetic acid. Tissues were mounted onto the glass slides for observation.

### Real-Time qRT-PCR

Inflorescences of wild-type (WT) and *SDS::SDS-BARNASE* independent *Arabidopsis* transgenic plants were collected for RNA isolation using the RNeasy Plant Mini Kit (Qiagen, Valencia, CA, USA). After determining the RNA quantification by the NanoDrop 2000c (Thermo Scientific, Bannockburn, IL, USA), RNA reverse transcription was conducted using the QuantiTect Reverse Transcription Kit (Qiagen, Valencia, CA, USA). Real-time PCR (DNA Engine Opticon 2 system, Hercules, CA, USA) and data analysis were performed as previously described (Liu et al., 2010a) to evaluate expressions of *A9, ATA7, DMC1*, and *SWI1* (Supplementary Table S1). The *ACTIN2* gene was used as an internal control. Three independent biological repeats were carried out.

### Microscopy

Pollen staining samples, GUS staining and semi-thin sections were observed and imaged with Olympus SZX7 and BX51 microscopes (Olympus, Center Valley, PA, USA), respectively. Images were obtained with an Olympus DP 70 digital camera (Olympus, Center Valley, PA, USA). For confocal microscopy analysis, anthers and ovules were dissected and mounted in water. The GFP signal was observed with a Leica TCS SP2 laser scanning confocal microscope (Leica, Buffalo Grove, IL, USA) using a 63×/1.4 water immersion objective lens. The 488-nm laser line was used to excite GFP and it also induced chlorophyll autofluorescence. The PMT gain settings was held at 650. GFP and chlorophyll autofluorescence were detected at 505–530 nm and 644–719 nm, respectively.

## Results

### *BARNASE* Driven by the 1.5-kb Promoter of the *SDS* Gene Caused Various Defects in Growth and Reproduction

**FIGURE 1:**
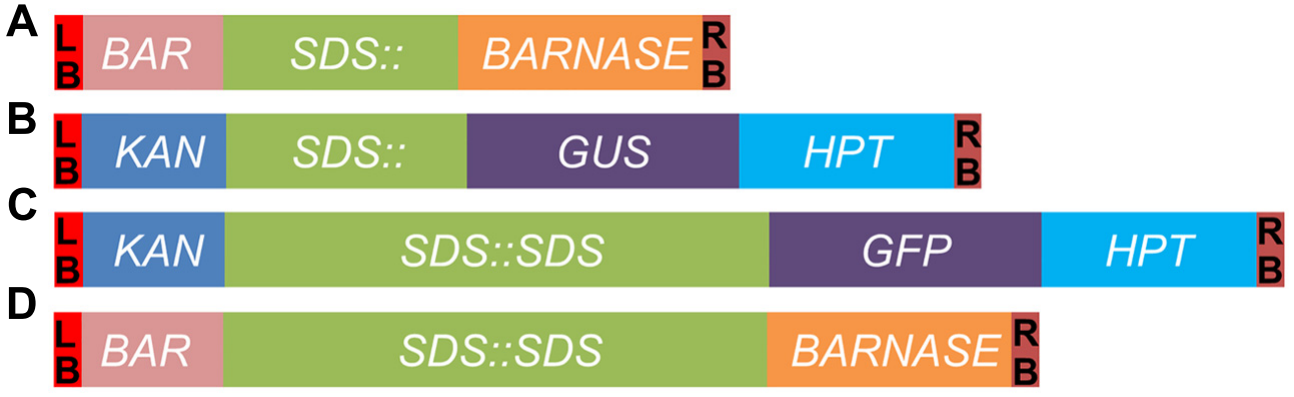
**Schematic diagrams of constructs generated in this study. (A)** *SDS::BARNASE*. **(B)** *SDS::GUS*. **(C)** *SDS::SDS-GFP*. **(D)** *SDS::SDS-BARNASE*. LB and RB, the T-DNA left and right border, respectively; *BAR*, the gene conferring resistance to the herbicide Basta; *SDS::*, the 1.5-kb promoter of the *SDS* gene; *BARNASE*, the bacterial ribonuclease; *KAN*, the kanamycin resistance gene; *GUS*, the gene encoding β-glucuronidase; *GFP*, the gene encoding green fluorescent protein; *HPT*, the hygromycin phosphotransferase gene; and *SDS::SDS*, the *SDS* genomic fragment containing a 1.5-kb promoter followed by a DNA fragment consisting of seven exons and six introns.

**FIGURE 2:**
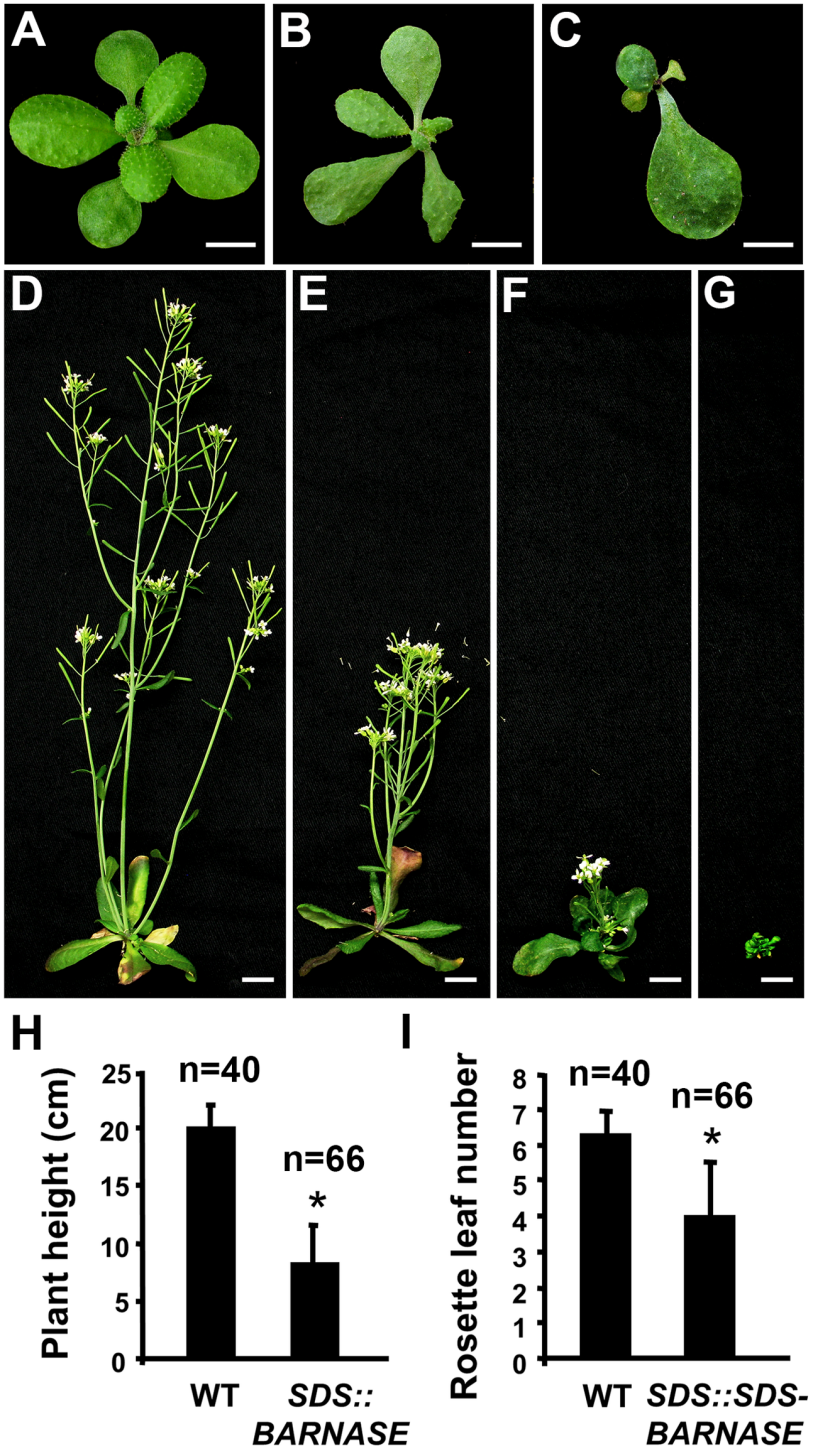
***SDS::BARNASE Arabidopsis* plants were abnormal in growth and development. (A–C)** Compared to wild type **(A)**, three-week old *SDS::BARNASE* **(B,C)** plants produced less rosette leaves with irregular shape. Bars = 0.5 cm. **(D–G)** Six-week old wild-type (WT, **D**) and *SDS::BARNASE* plants showing fertile but dwarf **(E)**, dwarf and sterile **(F)**, and no inflorescence **(G)** phenotypes. Bars = 1 cm. **(H)** Six-week old *SDS::BARNASE* plants were significantly shorter than the wild type. **(I)** The rosette leaf number of *SDS::BARNASE* adult plants was significantly reduced. “*n*” indicates the number of examined plants. Stars indicate significant difference (*P* < 0.01).

To create completely both male and female sterile plants without altering flower structure, we first generated the *SDS::BARNASE* construct using the 1.5-kb promoter of the *SDS* gene and a modified *BARNASE* (Zhang et al., 2012) to genetically ablate microspore and megaspore mother cells in *Arabidopsis* (**Figure 1A**). Among 66 examined *SDS::BARNASE* transgenic plants, none of them showed the specific phenotype in sterility. Instead, compared with the wild type (**Figure 2A**), *SDS::BARNASE* young plants were defective in vegetative growth, indicated by abnormal shape and numbers of rosette leaves (**Figures 2B,C**). Different from the WT adult plant (**Figure 2D**), *SDS::BARNASE* adult plants also exhibited various abnormal phenotypes, such as dwarf and fertile (**Figure 2E**), dwarf and sterile (**Figure 2F**), and even no inflorescence (**Figure 2G**). The height of mature *SDS::BARNASE* plants was significantly reduced (**Figure 2H**). Moreover, *SDS::BARNASE* plants produced significantly fewer rosette leaves than that of wild type (**Figure 2I**). Various defects of *SDS::BARNASE* plants in growth and development suggest that the 1.5-kb promoter of the *SDS* gene failed to drive the specific expression of *BARNASE* in microspore and megaspore mother cells.

### The 1.5 kb Upstream Region of the *SDS* Gene did not Confer its Meiocyte-Specific Expression

**FIGURE 3:**
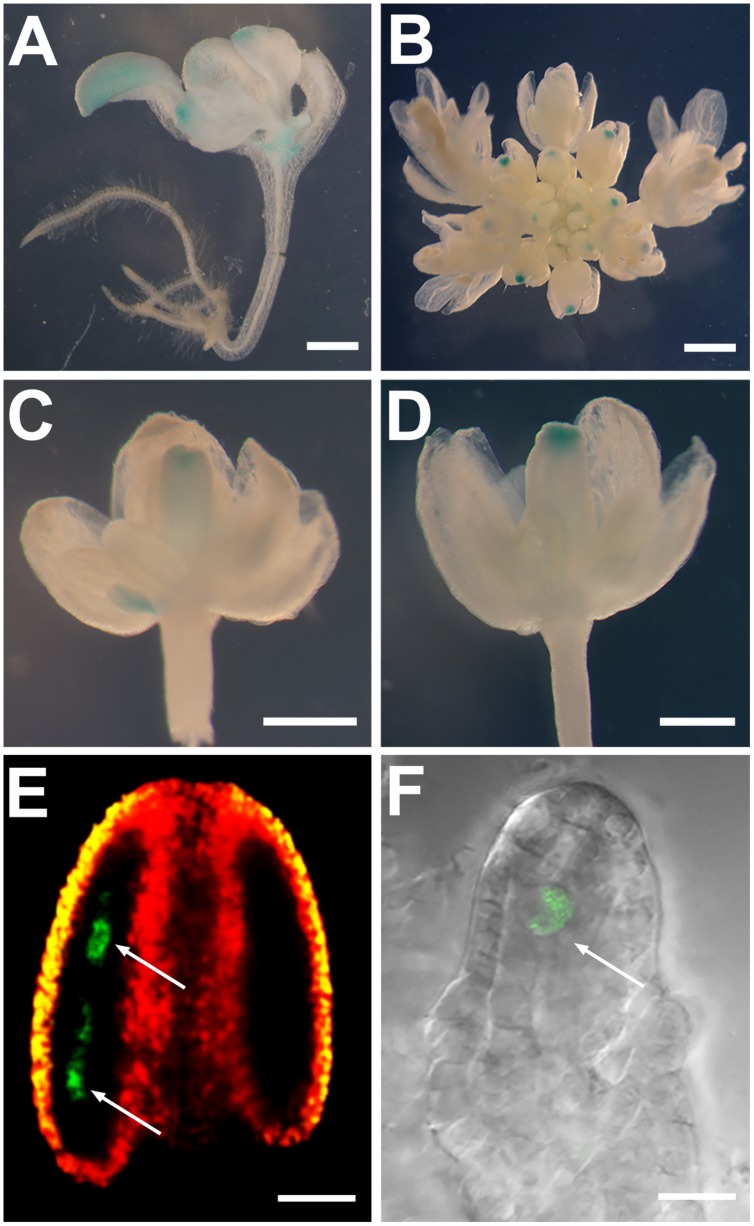
**The entire *SDS* gene but not the *SDS* 1.5-kb promoter confers the *SDS* meiocyte-specific expression. (A–D)** GUS staining of *SDS::GUS* plants showing GUS signals in cotyledons, true leaves, and shoot apical meristem of a young seedling **(A)**, as well as in carpels and stigmas of young buds **(B–D)**. **(E)** A confocal image from an *SDS::SDS-GFP* stage-5 anther showing the GFP signal (green color) only in microspore mother cells (arrows). Red and yellow colors showing merged autofluorescences. **(F)** A confocal image from an *SDS::SDS-GFP* stage 2-IV ovule showing the GFP signal only in the megaspore mother cell (arrow). Bars = 0.1 cm **(A,B)**, 0.5 mm **(C,D)**, 50 μm **(E)**, and 10 μm **(F)**.

Genetic ablation relies on the specificity of employed promoters. To examine why *BARNASE* under the control of the 1.5-kb *SDS* promoter did not achieve specific ablation effects on microspore and megaspore mother cells, we generated *SDS::GUS* plants to test the transcriptional activity of the 1.5-kb promoter (**Figure 1B**). Among 25 examined *SDS::GUS* transgenic plants, GUS signals were detected in cotyledons, true leaves, and shoot apical meristem of young seedlings (**Figure 3A**), as well as in carpels and stigmas of young buds (**Figures 3B–D**). Thus, our results suggest that the 1.5-kb promoter of the *SDS* gene was not sufficient for conferring its meiocyte-specific expression, which resulted in abnormal plant growth and development when it drove the expression of *BARNASE*.

### The Entire *SDS* Gene Led to the Meiocyte-Specific Expression of the SDS Protein

The possible existence of regulatory elements in *SDS* introns may contribute to the *SDS* meiocyte-specific expression. To test, how to achieve the specific expression of *SDS* in microspore and megaspore mother cells, we generated *SDS::SDS-GFP* constructs by fusing the *SDS* genomic fragment, containing the 1.5-kb promoter, seven exons and six introns, with the *GFP* gene (**Figure 1C**). In examined 18 *SDS::SDS-GFP* transgenic plants, we did not detect the GFP signal during the seedling stage and later in the vegetative growth stage. We, however, observed GFP signals only in microspore mother cells in anthers (**Figure 3E**) and megaspore mother cell in ovule during the reproductive stage (**Figure 3F**). Therefore, our results indicate that the entire *SDS* gene led to the meiocyte-specific expression of the SDS protein.

### *SDS::SDS-BARNASE* Caused Complete Both Male and Female Sterility but did not Affect Growth or Development in *Arabidopsis*

**FIGURE 4:**
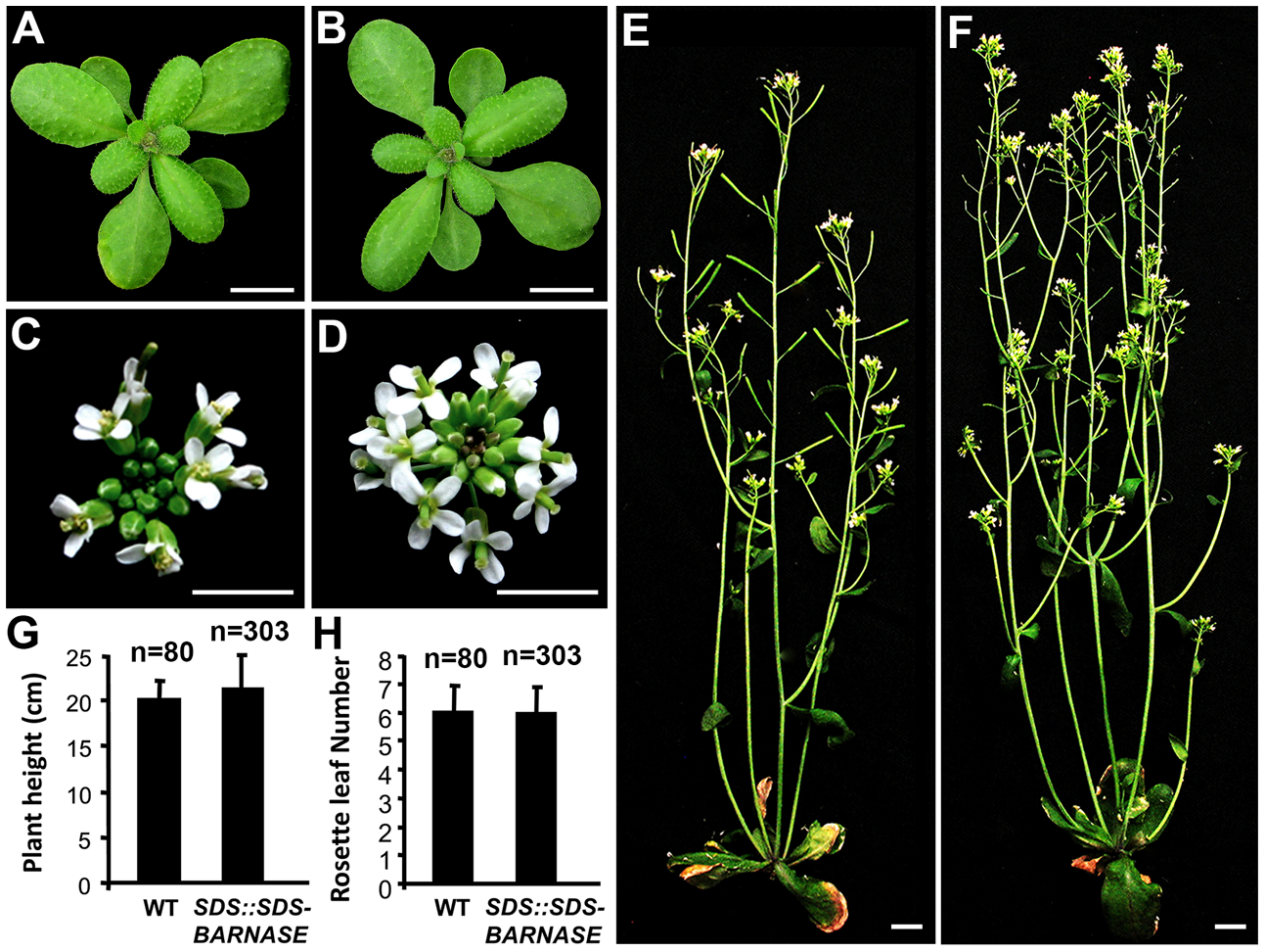
***SDS::SDS-BARNASE Arabidopsis* plants showed normal growth and development. (A,B)** Three-week old WT **(A)** and *SDS::SDS-BARNASE* **(B)** plants. Bars = 0.5 cm. **(C,D)** Five-week old WT **(C)** and *SDS::SDS-BARNASE* **(D)** inflorescences. Bars = 0.5 cm. **(E,F)** Six-week old WT **(E)** and *SDS::SDS-BARNASE* **(F)** plants. Bars = 1 cm. **(G)** No difference in average height between six-week old WT and *SDS::SDS-BARNASE* plants. **(H)** Similar rosette leaf numbers indicating no difference in flowering time between WT and *SDS::SDS-BARNASE* plants. “*n*” in **(G,H)** indicates the number of examined plants.

To generate complete both male and female sterility by specifically ablating microspore and megaspore mother cells, we made the *SDS::SDS-BARNASE* construct by fusing the *SDS* entire gene with the *BARNASE* gene (**Figure 1D**). We performed three transformations, resulting in 97, 80, and 126 *SDS::SDS-BARNASE* transgenic plants, respectively. All independent transgenic plants were sterile. We first evaluated the effects of *SDS::SDS-BARNASE* on growth and development. *SDS::SDS-BARNASE* transgenic plants produced rosette leaves with the same number, size, and shape as that of WT plants (**Figures 4A,B**). No morphological changes were observed in *SDS::SDS-BARNASE* inflorescences and flowers (**Figures 4C,D**). Moreover, mature *SDS::SDS-BARNASE* plants had a similar height to the wild type (**Figures 4E–G**). The flowering time of *SDS::SDS-BARNASE* plants was not affected either, because the same rosette leaf numbers as the wild type were produced when flowering (**Figure 4H**).

**FIGURE 5:**
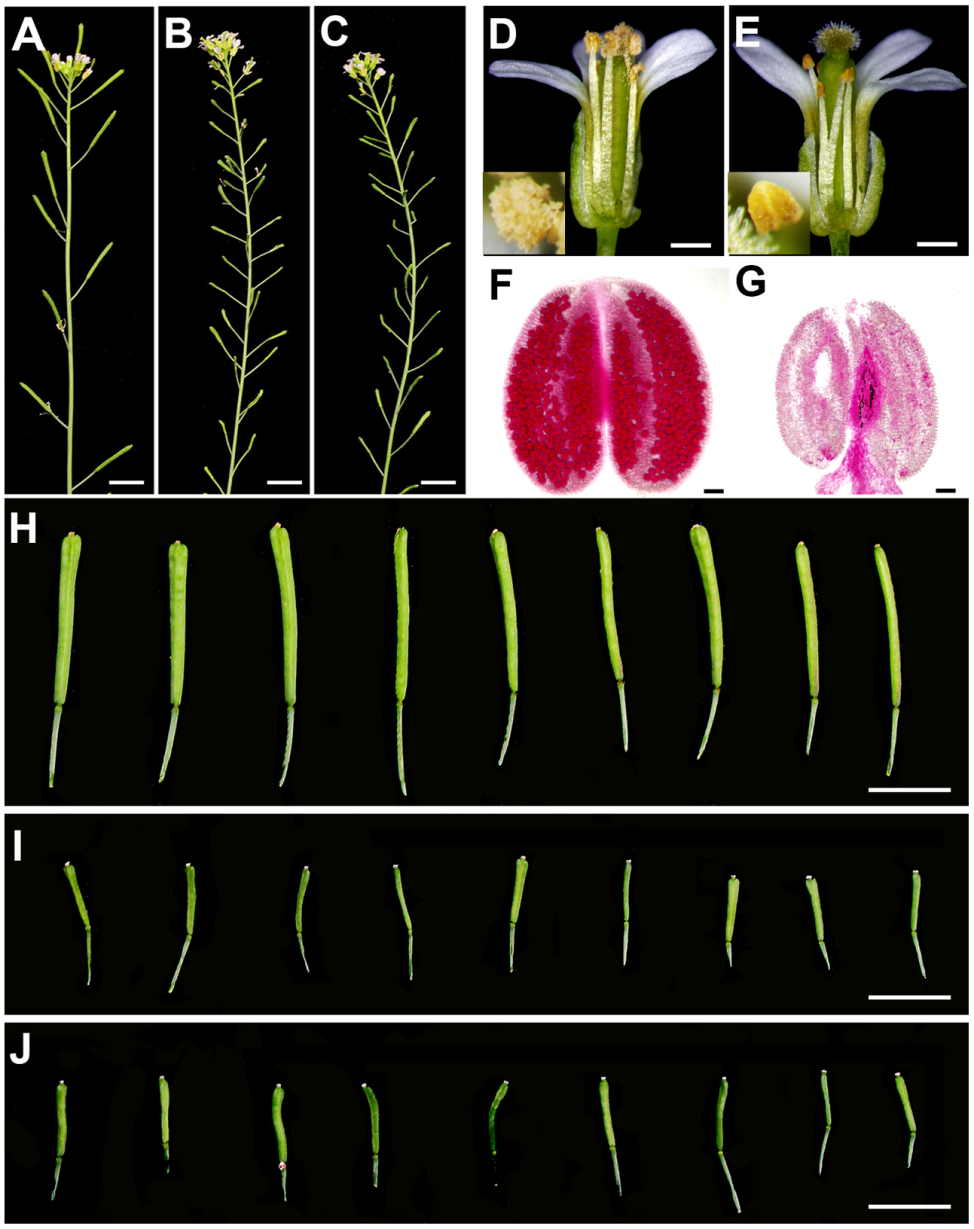
***SDS::SDS-BARNASE Arabidopsis* plants were completely both male and female sterile. (A–C)** Primary branches showing normal siliques in wild type **(A)** and short siliques indicating no developing seeds in *SDS::SDS-BARNASE* plants without **(B)** and with **(C)** pollination. Bars = 1 cm. **(D,E)** Side view of mature flowers (One sepal was removed, respectively) showing the *SDS::SDS-BARNASE* flower **(E)** is similar to the wild type **(D)** except short filaments. Pollen grains released from WT anthers (**D**, inset), while no pollen grains from *SDS::SDS-BARNASE* anthers (**E**, inset). Bars = 0.5 mm. **(F,G)** Pollen staining showing the WT anther full of viable pollen grains **(F)**, but no pollen grains from the *SDS::SDS-BARNASE* anther **(G)**. Bars = 30 μm. **(H–J)** Dissected individual siliques from primary inflorescences (positions 1–9) were long in wild type **(H)**, but short in *SDS::SDS-BARNASE* plants (**I**, without pollination; **J**, pollinated with WT pollen). Bars = 1 cm.

To further investigate sterility of *SDS::SDS-BARNASE* transgenic plants, we analyzed both male and female fertilities. Compared with the wild type (**Figures 5A,H**), *SDS::SDS-BARNASE* plants produced short siliques (**Figures 5B,I**). Except short filaments, *SDS::SDS-BARNASE* plants formed flowers that were the same as the wild type, indicated by four sepals, four petals, six stamens, and two carpels (**Figures 5D,E**). In the WT flower, pollen grains were released from anthers that reached the stigma (**Figure 5D**), whereas in the *SDS::SDS-BARNASE* flower, no pollen grains were observed on the anther surface and anthers did not reach the stigma (**Figure 5E**). Furthermore, different from the WT anther (**Figure 5F**), the *SDS::SDS-BARNASE* anther did not produce pollen grains (**Figure 5G**), indicating that *SDS::SDS-BARNASE* plants were male sterile. Because pollination using the WT pollen did not rescue the fertility (**Figures 5C,J**), *SDS::SDS-BARNASE* plants were female sterile too. Thus, using *SDS::SDS-BARNASE*, we efficiently created completely both male and female sterile *Arabidopsis* plants that had normal vegetative and reproductive growth and development, including the formation of all flower organs.

### *SDS::SDS-BARNASE* Inhibited Both Male and Female Gamete Formation

**FIGURE 6:**
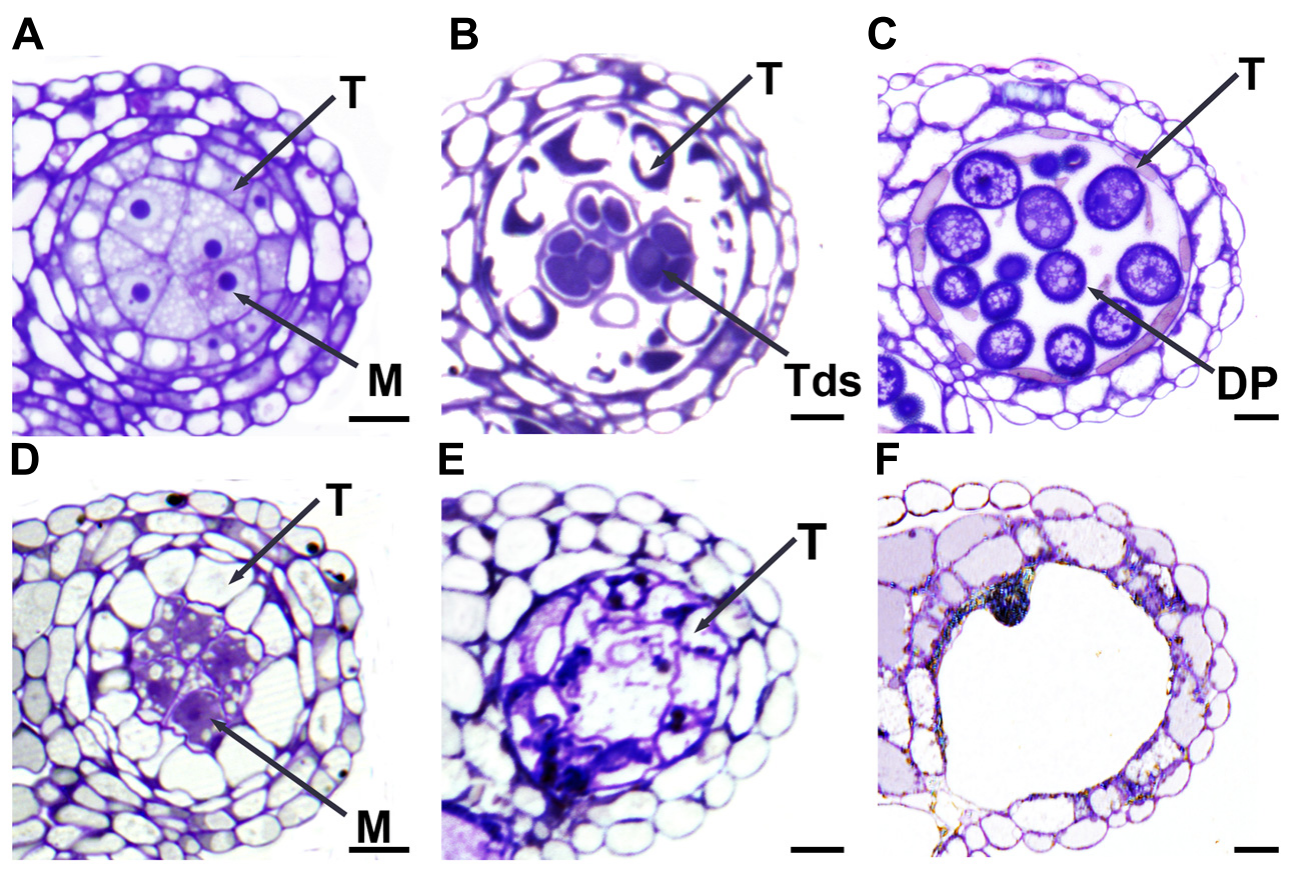
**Formation of male gametes was arrested in *SDS::SDS-BARNASE Arabidopsis* plants. (A–C)** WT anthers showing microsporocytes (microspore mother cells) and surrounding tapetal cells at stage 5 **(A)**, tetrads and tapetal cells at stage 7 **(B)**, and developing pollen grains at stage 9 **(C)**. **(D–F)** *SDS::SDS-BARNASE* anthers showing degenerating microsporocytes and precociously vacuolated tapetal cells at stage 5 **(D)**, dead microsporocytes and tapetal cells at stage 7 **(E)**, and a nearly empty anther lobe at stage 9 (only one dead pollen, **F**). M, microsporocytes (microspore mother cells); DP, developing pollen; T, tapetal cell; and Tds, tetrads.

To further understand ablation effects on microspore and megaspore mother cells, we did semi-thin sectioning of anthers and whole-mount squashes of ovules. At stage 5 (Sanders et al., 1999; Zhao et al., 2002), when compared with the WT anther (**Figure 6A**), the *SDS::SDS-BARNASE* anther showed vacuolated microsporocytes (microspore mother cells) and tapetal cells (**Figure 6D**), indicating the degeneration of both cells. At stage 7 in the WT anther, successful male meiosis resulted in the formation of tetrads (**Figure 6B**), whereas in the *SDS::SDS-BARNASE* anther, tetrads, and tapetal cells were collapsed (**Figure 6E**). At stage 9, the WT anther contains developing pollen grains (**Figure 6C**), but the *SDS::SDS-BARNASE* anther lacked developing microspores (**Figure 6F**).

**FIGURE 7:**
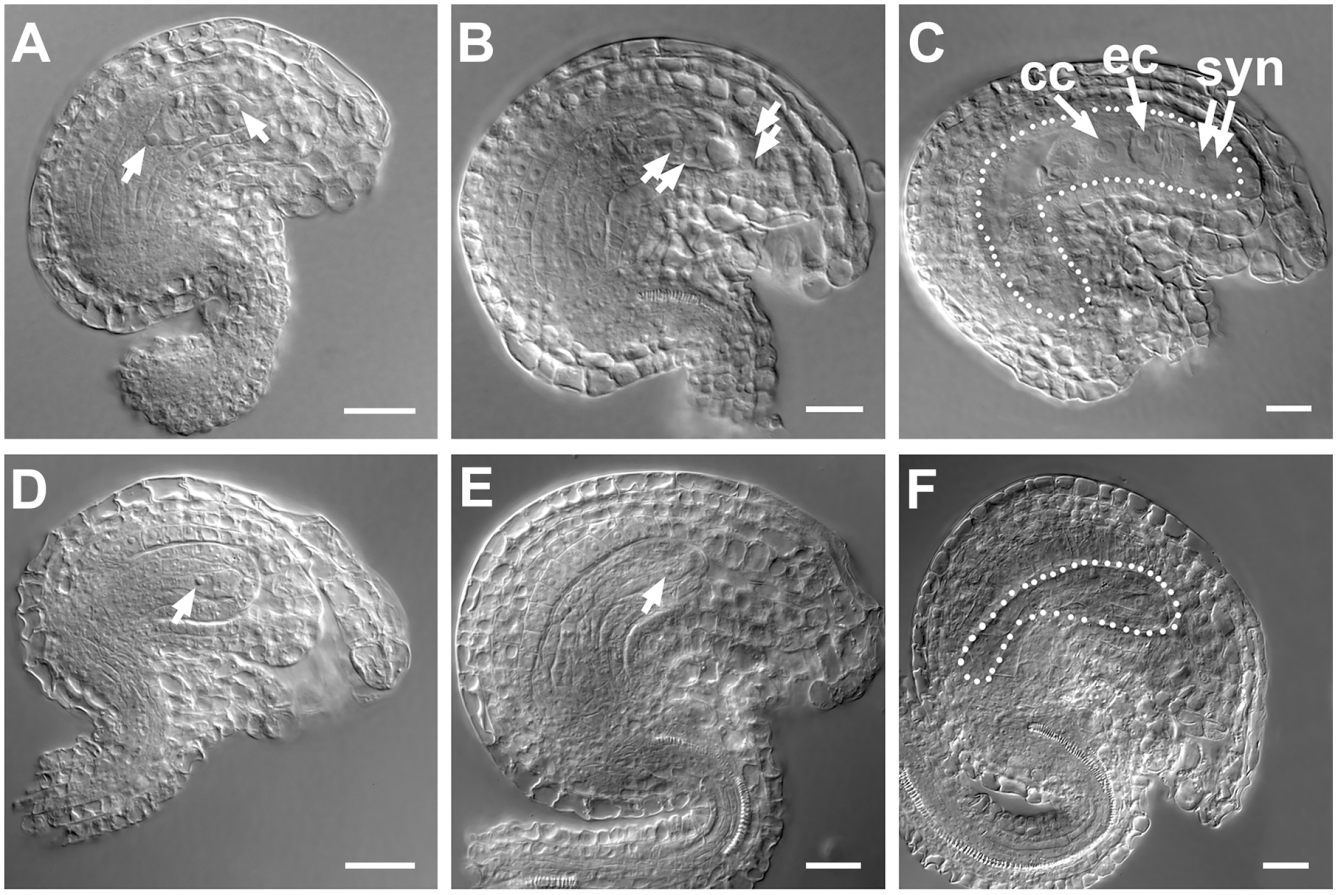
**Formation of female gamete was arrested in *SDS::SDS-BARNASE Arabidopsis* plants. (A–C)** WT ovules showing two separated nuclei (arrows) at the FG3 stage **(A)**, four nuclei (arrows) at the FG4 stage **(B)**, and the central cell, the egg cell, and synergid cells in a mature embryo sac (white dots outlined) at the FG6 stage **(C)**. **(D–F)** *SDS::SDS-BARNASE* ovules showing one small nucleus (arrow) at both FG3 **(D)** and FG4 **(E)** stages and a small empty embryo sac (white dots outlined) at the FG6 stage **(F)**. Bars = 10 μm. cc, central cell; ec, egg cell; and syn, synergid cells.

In embryo sacs of WT ovules, two nuclei at stage FG3 (Pagnussat et al., 2009) (**Figure 7A**) and four nuclei at stage FG4 (**Figure 7B**) were observed; however, in *SDS::SDS-BARNASE* embryo sacs, only a single nucleus was produced (**Figures 7D,E**). At stage FG6, the WT embryo sac showed the central cell, the egg cell, and synergid cells (**Figure 7C**), but the *SDS::SDS-BARNASE* embryo sac is empty (**Figure 7F**).

**FIGURE 8:**
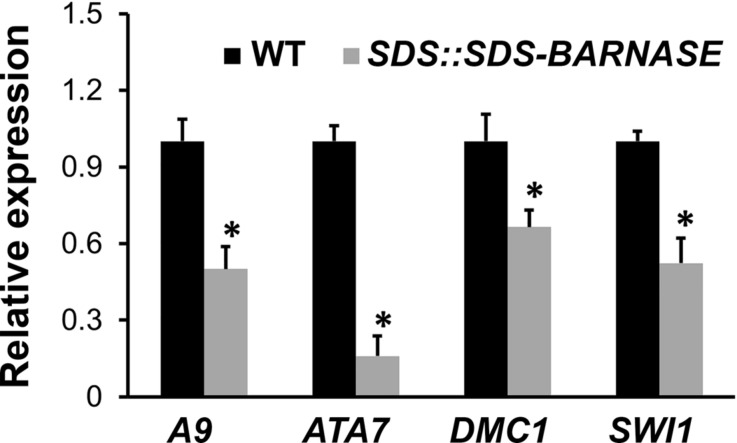
**Expressions of tapetal cell as well as microspore and megaspore mother cell marker genes.** Real-time qRT-PCR showing decreased expressions of tapetal cell marker genes *A9* and *ATA7* as well as microspore and megaspore mother cell marker genes *DMC1* and *SWI1*. Stars indicate significant difference (*P* < 0.01).

Furthermore, our results showed that expressions of tapetal cell marker genes *A9* and *ATA7* as well as microspore and megaspore mother cell marker genes *DMC1* and *SWI1* were significantly decreased in *SDS::SDS-BARNASE* buds in comparison to the wild type (**Figure 8**). In summary, the specific expression of the SDS-BARNASE toxic fusion protein in microspore and megaspore mother cells efficiently impaired the production of both male and female gametes, which led to absolute both male and female sterility, but did not affect flower organ formation or plant growth and development.

### *SDS::SDS-BARNASE* Caused Complete Both Male and Female Sterility but did not Affect Growth or Development in Tobacco

**FIGURE 9:**
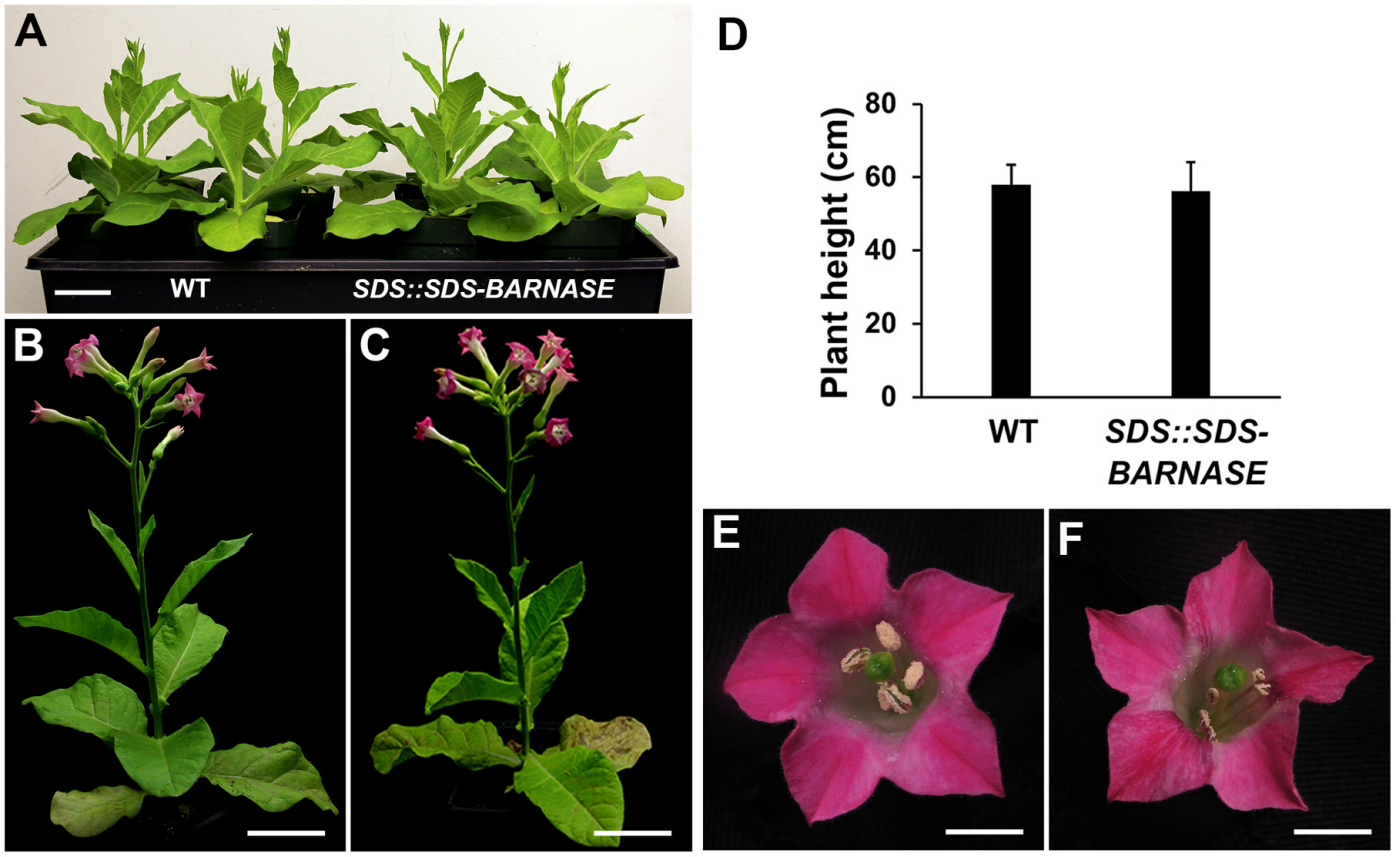
***SDS::SDS-BARNASE* tobacco plants showed normal growth and development. (A)** Forty-day old tobacco WT and *SDS::SDS-BARNASE* plants. Bar = 5 cm. **(B,C)** Sixty-day old WT **(B)** and *SDS::SDS-BARNASE* **(C)** plants. Bars = 10 cm. **(D)** No difference in average height between WT and *SDS::SDS-BARNASE* adult plants. **(E,F)** Flower size, color, and structure remained the same in WT and *SDS::SDS-BARNASE* plants. Bars = 1 cm.

To test whether *SDS::SDS-BARNASE* can provide a general tool to create both male and female sterile plants, we transformed it into tobacco and generated *SDS::SDS-BARNASE* tobacco transgenic plants by tissue culture. Among 14 examined *SDS::SDS-BARNASE* tobacco transgenic lines, leaf shape and size (**Figures 9A–C**), as well as the plant height (**Figures 9B–D**) were the same as that of WT plants. In addition, the *SDS::SDS-BARNASE* tobacco flower had the same size, color, and structure as that of wild type (**Figures 9E,F**). Therefore, *SDS::SDS-BARNASE* did not affect growth or development in tobacco plants.

**FIGURE 10:**
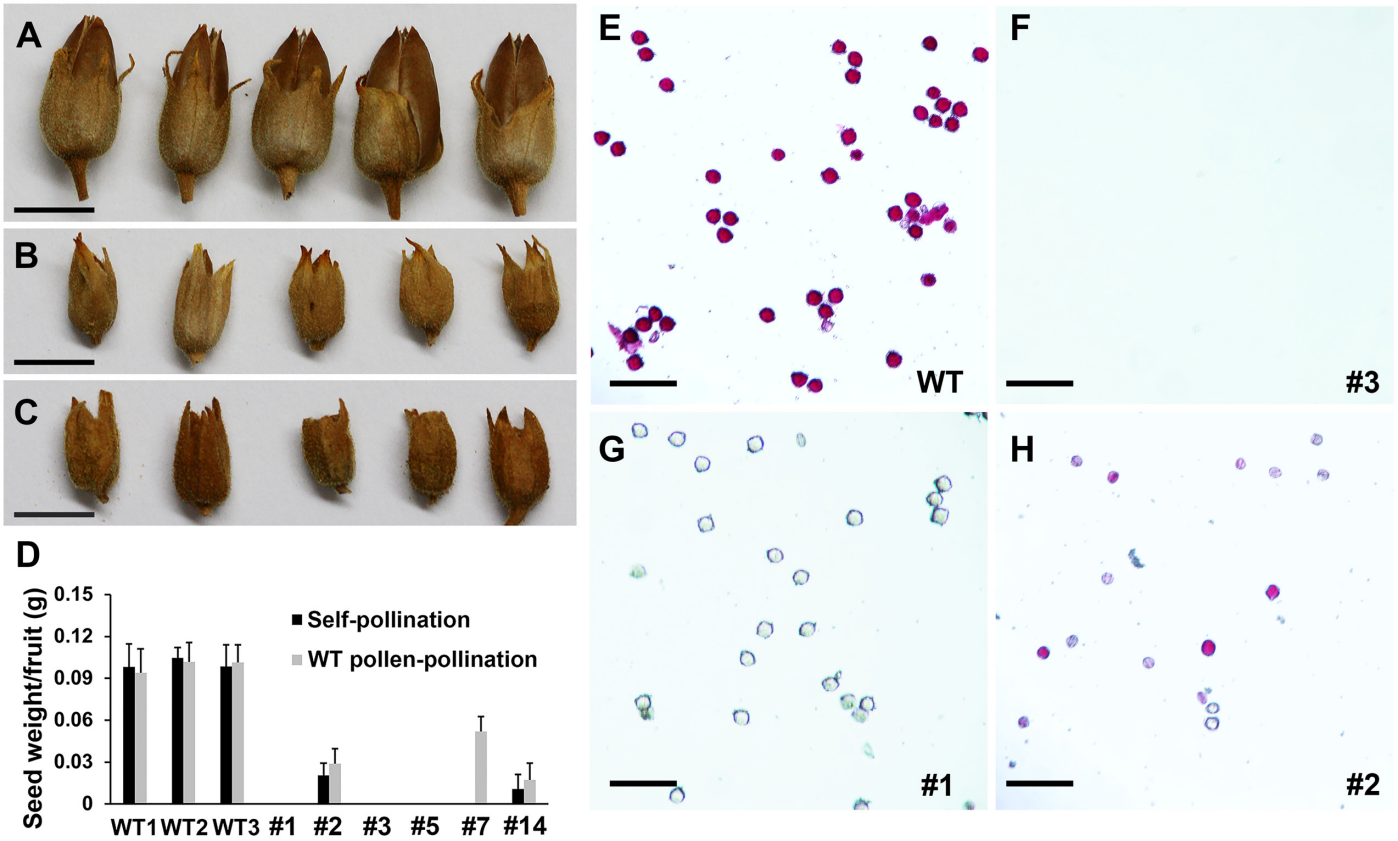
***SDS::SDS-BARNASE* tobacco plants were completely both male and female sterile. (A–C)** Large fruits from the WT plant **(A)** and small fruits from *SDS::SDS-BARNASE* plants without **(B)** and with **(C)** manual pollination with WT pollen grains. Bars = 1 cm. **(D)** The weight of seeds per self-pollinated and manually pollinated fruit (*n* = 5), respectively. Numbers indicate examined independent transgenic lines. **(E)** WT viable pollen grains in red color. **(F–H)** no **(F)**, all dead **(G)** and a few viable **(H)** pollen grains in *SDS::SDS-BARNASE* plants. Numbers indicate examined independent transgenic lines. Bars = 100 μm.

Ten examined *SDS::SDS-BARNASE* tobacco transgenic lines were completely sterile. WT tobacco plants produced large fruits and per fruit averagely contained 0.11 g of seeds (**Figures 10A,D**). Conversely, *SDS::SDS-BARNASE* plants produced small fruits and no seeds were found when self-pollenated (**Figures 10B,D**, e.g., plants #1, 3, 5, and 7). Further pollen viability analysis showed that WT tobacco anthers produced viable pollen, indicated by red color (**Figure 10E**), whereas anthers from sterile tobacco plants either lacked pollen grains (**Figure 10F**) or formed dead pollen grains (**Figure 10G**). The four non-absolutely sterile lines produced a few seeds (**Figure 10D**, e.g., plants #2, and 14) and only some functional pollen grains were found in anthers of those lines (**Figure 10H**, e.g., plant #2). Our results suggest that *SDS::SDS-BARNASE* impaired male fertility in tobacco.

We then examined the female fertility in sterile tobacco transgenic plants. The fertility of manually male-sterilized WT flowers could be rescued by cross-pollination with WT pollen (**Figure 10D**), but following cross-pollination with WT pollen, the fruit size of *SDS::SDS-BARNASE* sterile tobacco plants did not change (**Figure 10C**) and no seeds were produced (**Figure 10D**, e.g., plants #1, 3, and 5). Thus, *SDS::SDS-BARNASE* tobacco transgenic plants were also female sterile. Manual pollination partially rescued the fertility of line #7, indicating that the line #7 is a completely male but partially female sterile plant, while lines #2 and 14 were nearly male and female sterile plants (**Figure 10D**). Collectively, a majority of *SDS::SDS-BARNASE* tobacco transgenic plants were completely male and female sterile, suggesting that *SDS::SDS-BARNASE* is functionally conserved, which can be used to create both male and female sterility in general.

## Discussion

*SOLO DANCERS*, a unique type of (SDS-type) meiosis-specific cyclin, is conserved in flowering plants (Wang et al., 2004; Zhang et al., 2014; Wu et al., 2015). In our studies, the 1.5-kb *SDS* promoter did not achieve the specific expression of *SDS* in either microspore or megaspore mother cells. Conversely, the entire *SDS* gene genomic fragment did. The intron-dependent spatial expression has been revealed in different genes from various species, including *SUS3* and *SUS4* in potato (Fu et al., 1995a,b), *OsTubA1* in rice (Jeon et al., 2000; Giani et al., 2009), *PhADF1* in petunia (Jeong et al., 2007), as well as *AGAMOUS*, *ACT1* and *PRF* in *Arabidopsis* (An et al., 1996; Sieburth and Meyerowitz, 1997; Jeong et al., 2006). Therefore, it is possible that regulatory motifs in *SDS* introns contribute to its specific spatial and temporal expression. Future studies should be focused on dissecting the functions of unknown regulatory motifs and then making a synthetic promoter that confers the strong and specific expression of *SDS* in microspore and megaspore mother cells. We found a few non-completely sterile tobacco transgenic plants, suggesting that the *Arabidopsis SDS* gene did not work efficiently in tobacco. In order to achieve accurate and efficient ablation effects, it would be more practical to use *SDS* orthologous genes of target species or the synthetic promoter to drive *BARNASE*.

The existing methods for creating male sterile only GM plants are not able to completely prevent transgene flow, because pollen from non-GM plants can rescue seed development. In addition, current sterilization technologies either suppress the production of entire flower or some floral organs, which may cause potential ecological effects besides transgene flow. Furthermore, BARNASE and DTA are very toxic. Many “specific” promoters still have basal activities in other organs; therefore, the low expression of BARNASE/DTA in non-target organs often reduces plant survival rate and inhibits plant growth. Microspore and megaspore mother cells are two small groups of male and female reproductive cells, which are differentiated after all floral organs are established. Ablating microspore and megaspore mother cells only leads to elimination of male and female gametes, but it does not affect other somatic cell differentiation and flower development. In this study, we specifically ablated microspore and megaspore mother cells using the SDS and BARNASE fusion protein. Thus, our research developed an efficient strategy to successfully create completely both male and female sterile plants; however, the plant growth and development, including the formation of all flower organs, were not affected.

Genetically modified crops have been widely cultivated in many countries due to their improved agronomic traits; however, the adoption of GM trees (e.g., poplar, eucalypts, and pines) and perennial grasses (e.g., miscanthus and switchgrass) is limited, because those plants are long-lived, weakly domesticated, and important to ecosystems. Various studies has been done to increase cold tolerance and biomass, modify lignin and cellulose biosynthesis, or alter growth and flowering of *Eucalyptus* (Girijashankar, 2011; Hinchee et al., 2011; Klocko et al., 2015), aspen (Etchells et al., 2015), poplar (Wilkerson et al., 2014), and switchgrass (Fu et al., 2011; Shen et al., 2013; Baxter et al., 2014; Poovaiah et al., 2015). Our research developed a general and effective approach to create completely both male and female sterile plants by specifically ablating microspore and megaspore mother cells, which provides a solution for overcoming regulatory hurdles to field research and ultimately commercial uses of GM plants.

Besides the transgene containment, our method can be applied for modifying invasive and ornamental plants. Male and female sterilized invasive plants generated by our method can be planted for multiple purposes, such as forestry and horticulture. The main valuable trait for many ornamental trees, such as cherries and plums, is the beauty of flowers. Fruits often make the garden messy. Moreover, nutrient competition from fruit setting affects flower organ differentiation, and consequently reduces flower numbers in the coming year. Our new method serves as an excellent tool to engineer ornamental trees that still produce attractive intact flowers without fruit setting, which, therefore, maintains their ornamental value.

## Author Contributions

DZ conceived and designed the experiments. JH, AS, and TZ performed experiments. All authors analyzed data. DZ and JH wrote the article.

## Conflict of Interest Statement

The authors declare that the research was conducted in the absence of any commercial or financial relationships that could be construed as a potential conflict of interest. Dazhong Zhao has filed a patent application for the sterilization technique reported in this article.
