# Supplementary material for: Creating Completely Both Male and Female Sterile Plants by Specifically Ablating Microspore and Megaspore Mother Cells
Source: Front Plant Sci. 2016 Feb 1;7:30. doi: 10.3389/fpls.2016.00030 (PMC4740954; doi:10.3389/fpls.2016.00030)
Supplement: Supplementary file 1 [file Table_1.DOC]

**Supplementary Material**

**Creating completely both male and female sterile plants by specifically ablating microspore and megaspore mother cells**

Jian Huang, Ashley R. Smith, Tianyu Zhang and Dazhong Zhao

Department of Biological Sciences, University of Wisconsin-Milwaukee, Milwaukee, WI, USA

| **Supplemental Table S1**. Primers used in this study. | | | | |
| --- | --- | --- | --- | --- |
| Primer ID | Primer name | Purpose | Enzyme digestion site | Sequence (5' to 3') |
| zp1283 | *SDS* promoter 5' | pENTR-*SDS* | Kpn I | CACCGGTACCCCATCATTCTCGTCT CTCTCGCAC |
| zp1284 | *SDS* promoter 3' | pENTR-*SDS* | BsrGI | CAGTGTACATTTTTCTCCGTACGAA AGCTTGAAAC |
| zp1823 | *mGFP5er* 5' | pEarleyGate303-  *mGFP5er* | XhoI | CCGCTCGAGGCAGGCTTTATGAAG AC |
| zp1824 | *mGFP5er* 3' | pEarleyGate303-  *mGFP5er* | XbaI | GCTCTAGAGCGGCCGCCGATCTAG TAAC |
| zp1768 | *BARSTAR* 5' | pCR2.1-*BARSTAR* | NsiI | CCAATGCATTGGCGTATAACATAG  TATCGAC |
| zp1769 | *BARSTAR* 3' | pCR2.1-*BARSTAR* | NsiI | CCAATGCATATGGCAGCGCTGGCA  GTC |
| zp1770 | XhoI 5' | pEarleyGate303-  *BARSTAR(XhoI)* | BglII | GAAGATCTGGATCCGGCTTAC |
| zp1771 | XhoI 3' | pEarleyGate303-  *BARSTAR(XhoI)* | XbaI, XhoI | GCTCTAGACTCGAGCTGTTCCACC  ACTTTGTAC |
| zp1772 | *BARNASE* 5' | pEarleyGate303-  *BARSTAR-BARNASE* | XhoI | CCGCTCGAGTACGCTGTGAGGATC TGTG |
| zp1773 | *BARNASE* 3' | pEarleyGate303-  *BARSTAR-BARNASE* | XbaI | GCTCTAGAAGGATATCCTGATCCG TTGAC |
| zp2163 | *SWI1* 5' | Real-time PCR |  | GGAGGAAGACATGGGATGGC |
| zp2164 | *SWI1* 3' | Real-time PCR |  | CCCTTGTTCACCACCTTCACTTC |
| zp2165 | *DMC1* 5' | Real-time PCR |  | GGAGAACTCGCAGACCGCC |
| zp2166 | *DMC1* 3' | Real-time PCR |  | CCACCTGGGTCAGCTATGAC |
| zp1196 | *A9* 5' | Real-time PCR |  | ATGGTATCTCTAAAGTCCCTTG |
| zp1197 | *A9* 3' | Real-time PCR |  | CCAAATCCTCGGAACTGAATG |
| zp851 | *ATA7* 5' | Real-time PCR |  | CGTCTCCAGGATCGAGGAAT |
| zp852 | *ATA7* 3' | Real-time PCR |  | GGAGATGGGAAAGCTGAGAG |
| zp853 | *ACTIN2* 5' | Real-time PCR |  | GTTGGGATGAACCAGAAGGA |
| zp854 | *ACTIN2* 3' | Real-time PCR |  | GAGGAGCCTCGGTAAGAAGA |
